# White Matter Abnormalities and Animal Models Examining a Putative Role of Altered White Matter in Schizophrenia

**DOI:** 10.1155/2011/826976

**Published:** 2011-08-11

**Authors:** Haiyun Xu, Xin-Min Li

**Affiliations:** ^1^Department of Anatomy, Southern Illinois University Carbondale, Carbondale, IL 62901, USA; ^2^Department of Psychiatry, Faculty of Medicine, University of Manitoba, Winnipeg, MB, Canada R3T 2N2

## Abstract

Schizophrenia is a severe mental disorder affecting about 1% of the population worldwide. Although the dopamine (DA) hypothesis is still keeping a dominant position in schizophrenia research, new advances have been emerging in recent years, which suggest the implication of white matter abnormalities in schizophrenia. In this paper, we will briefly review some of recent human studies showing white matter abnormalities in schizophrenic brains and altered oligodendrocyte-(OL-) and myelin-related genes in patients with schizophrenia and will consider abnormal behaviors reported in patients with white matter diseases. Following these, we will selectively introduce some animal models examining a putative role of white matter abnormalities in schizophrenia. The emphasis will be put on the cuprizone (CPZ) model. CPZ-fed mice show demyelination and OLs loss, display schizophrenia-related behaviors, and have higher DA levels in the prefrontal cortex. These features suggest that the CPZ model is a novel animal model of schizophrenia.

## 1. Introduction

Schizophrenia is a devastating mental disorder affecting about 1% of the population worldwide [1]. The onset of schizophrenia ranges from mid to late adolescence through early adulthood; the majority of cases occur between the ages of 16 and 30 years [2]. Clinically, this disorder is characterized by positive symptoms (psychosis, hallucinations, and paranoia), negative symptoms (flat affect, poor attention, lack of motivation, and deficits in social function), and cognitive deficits. 

The positive symptoms of schizophrenia have been treatable since chlorpromazine was introduced into clinical practice in the early 1950s. Since then, a number of antipsychotic drugs have been developed, which are grouped into typical and atypical antipsychotics. All typical antipsychotics have high affinities for dopamine (DA) D_2_  receptors, which correlate with the therapeutic doses of these drugs [3–6]. These observations, plus the psychotogenic effects of DA-enhancing drugs [7, 8], provided solid evidence for the DA hypothesis of schizophrenia that hyperactivity of DA transmission is responsible for the positive symptoms observed in this disorder [9].

In contrast to positive symptoms, negative symptoms tend to remain stable over time in patients with established illness [10] and have been found to persist despite of treatment [11, 12]. This phenomenon raised a critical challenge to the DA hypothesis. In addition, this hypothesis cannot account for why the symptoms of schizophrenia commonly first present in late adolescence and early adulthood. 

During adolescence and early adulthood, white matter volume expands while grey matter volume loss occurs [13]. This long-lasting development of the white matter is associated with the development of cognitive functions [14]. Given these and that schizophrenia present in adolescence or early adulthood, it is reasonable to infer that disruption to white matter development and/or damage to some white matter structures during this period are responsible for the development of psychotic symptoms. In line with this view, there are increasing human studies, especially those following the inspiring review by Davis et al. [15], showing white matter abnormalities and altered oligodendrocyte- (OL) and myelin-related genes in schizophrenic patients. On the other hand, abnormal behaviors are reported in patients with white matter diseases. In this paper, we will briefly review some of these human studies. Following that, we will selectively introduce some animal models that examine a putative role of white matter abnormalities in schizophrenia. The emphasis will be put on the cuprizone-fed mouse, a novel animal model of schizophrenia. 

## 2. White Matter Abnormalities in Schizophrenic Patients

### 2.1. Imaging Evidence

Lateral ventricular enlargement is the best replicated anatomic abnormality detected in the brains of patients with schizophrenia both in earlier computed tomography (CT) studies and in many magnetic resonance imaging (MRI) investigations [16]. The boundaries of the cerebral ventricles are largely made up of white matter structures. Therefore, ventricular enlargement may be due in part to volumetric reduction of adjacent white matter tracts. However, conventional MRI findings in earlier studies for cerebral white matter volume in schizophrenia have been mixed. Some of them found no differences in white matter volume between schizophrenic patients and normal subjects [17–21], while some reported white matter reductions in schizophrenia [22–24].

Foong's group [25] for the first time used magnetization transfer imaging (MTI), a technique sensitive to myelin and axonal abnormalities, to investigate the white matter in patients with schizophrenia. They found that the magnetization transfer ratios (MTRs) significantly reduced in the right and left temporal regions in schizophrenic patients compared with controls. The same group also used diffusion tensor imaging (DTI), another new MRI technique capable of examining water diffusion in different tissues and the organization of white matter tracts, to investigate the neuropathology of the corpus callosum in patients with schizophrenia [26]. The mean diffusivity (MD) increased and fractional anisotropy (FA) reduced in the splenium of the corpus callosum in the schizophrenic group compared with controls. These results confirmed the findings in an earlier study, which reported a reduction in FA in the corpus callosum in a small group of schizophrenic patients [27].

A number of recent DTI studies in patients with schizophrenia [28–33] reported FA reduction in various brain regions/structures, including the frontal white matter, the deep frontal perigenual region, the medial occipital lobe, the inferior parietal gyri, the middle temporal gyri, the parahippocampal gyri, the corpus callosum, the internal capsule, the cingulum bundle, the fornix, the superior occipitofrontal fasciculus, the frontal longitudinal fasciculus, the right inferior occipitofrontal fasciculus, the right medial temporal lobe adjacent to the right parahippocampal gyrus, the left arcuate fasciculus, the left superior temporal gyrus, and the left uncinate fasciculus. Moreover, significant FA reductions were reported in all white matter regions bilaterally in schizophrenic patients of a recent study [34]. Lower FA values were also seen in never-medicated, first-episode schizophrenia [35–38]. More significantly, white matter abnormalities in specific brain regions were associated with different dimensions of schizophrenia symptoms. For example, widespread decrements in prefrontal white matter in schizophrenic patients were related to higher levels of negative symptoms, as measured by the Scale for the Assessment of Negative Symptoms (SANS) [39]; inferior frontal white matter FA inversely correlated with the SANS global ratings of negative symptoms [40]; a significant reduction of white matter in parietal cortex of right hemisphere was found in a subgroup of patients with pronounced negative symptoms [41]. In a more recent study, there were significant positive correlations between volumes (larger) in anterior callosal, cingulate and temporal deep white matter regions and positive symptoms, such as hallucinations, delusions and bizarre behavior. Negative symptoms were negatively related to volumes (smaller) in occipital and paralimbic superficial white matter and posterior callosal fiber systems [42]. 

### 2.2. Postmortem Evidence

Decreased (MTR) found in schizophrenic patients suggests decreases in the myelin or axonal membrane integrity. Similarly, FA decrease reflects the reduction in the coherence of white matter. These suggestions are in line with the results from postmortem studies, exemplified by reduced myelin basic protein (MBP) immunoreactivity [43] and myelin pallor and myelin loss [44] in brains of chronic schizophrenic patients. Moreover, ultrastructural evidence of reduced myelin sheath compactness, including bodies and lamellar bodies, has been shown in postmortem electron microscopy studies [45–47]. In addition, a delayed myelination in the PFC was reported in patients with schizophrenia, suggesting the developmental nature of this change [48]. 

OLs are myelin sheath producing cells. Therefore, myelin sheath changes reflect OLs alterations. Indeed, OLs were first implicated in schizophrenia in 1938 when swollen OLs were observed in schizophrenic brains postmortem [49]. In recent years, the group of Uranova and Orlovskaya carried out a series of postmortem studies and reported solid evidence of disturbed structure and function of OLs [45, 46, 50–53], found reductions in density and size of OLs in prefrontal and striatal regions [52, 54, 55], and showed a deficit of OLs in PFC and adjacent white matter [56] in schizophrenia. Loss and altered spatial distribution of OLs were also found in the superior frontal gyrus in schizophrenia [57]. 

## 3. Altered OL- and Myelin-Related Genes in Schizophrenia

In a groundbreaking study, Hakak et al. [58] found that the expression of a series of genes related to OLs and myelin significantly decreased in the dorsolateral prefrontal cortex (DLPFC) samples of schizophrenic patients. Another microarray study measured expression of approximately 12,000 genes in the middle temporal gyrus and found significant decreases in the expression of some myelin-related genes in subjects with schizophrenia [59]. In a more recent study [60], variations in myelin- and OL-related gene expression were found in multiple brain regions of postmortem schizophrenic brains. The downregulated myelin- and OL-related genes include neuregulin 1(*NRG1*), CNP (2′, 3′-cyclic nucleotide 3′-phosphodiestase), CLDN11 (claudin 11, an OL specific protein), OLIG2 (OL lineage transcription factor 2), MAG (myelin associated glycoprotein), MAL (myelin and leukocytes protein), OKI (quaking homolog, KH domain RNA binding (mouse)), TM4SF11 (trans-membrane 4 superfamily 11), and GELS (gelsolin). In the following, we will selectively review evidence for changes in some of the above mentioned genes. 

### 3.1. NRG1

This is a family of proteins containing an epidermal growth factor-like domain that specifically activate receptor tyrosine kinases of the erbB family: erbB2, erbB3, and erbB4 [61]. NRG1-mediated erbB signaling has important roles in neural and glial development, as well as in the regulation of neurotransmitter receptors thought to be involved in the pathophysiology of schizophrenia. In the study by Hakak et al. [58], a significant reduction in the level of e*rbB3* expression was found in PFC of schizophrenic patients. This decrease was confirmed by quantitative and differential-display RT-PCR analysis [62]. In a genome-wide scan, Stefansson et al. [63], by means of haplotype analysis, identified *NRG1* as a candidate gene for schizophrenia. This association of *NRG1* with schizophrenia was confirmed by the same group in a Scottish population [64] and by an independent study in a large sample of unrelated Welsh patients [65]. Strong association between *NRG1* and schizophrenia was also found in Chinese population [66, 67], but not in Japanese population [68]. In a more recent study [69], schizophrenic patients with the T allele for a single-nucleotide polymorphism (SNP8NRG221533) showed significantly decreased anterior cingulum FA compared with patients homozygous for the C allele and healthy controls who were T carriers, suggesting that *NRG1* variation may play a role in the white matter abnormality of this brain region in patients with schizophrenia. 

### 3.2. CNP

This gene maps to 17q21.2, a region of the genome in which genome-wide evidence for linkage to schizophrenia was observed in a single pedigree [65]. CNP (protein) can be detected early in development, in the precursor cells to OLs. In adulthood, CNP shows a high turnover compared with other myelin-associated proteins [70]. Postmortem studies of anterior frontal cortex demonstrated less immunoreactivity of CNP in schizophrenia [71]. This result confirmed the downregulation of *CNP* gene in the schizophrenic brain [58]. Compatible with the underexpression of *CNP *mRNA in schizophrenia, the low-expressing A allele was significantly associated with schizophrenia in a case-control sample. All affected individuals in the linked pedigree were homozygous for the low-expression allele [72]. In a recent human study, reduced CNP protein in the hippocampus and anterior cingulated cortex of patients with schizophrenia was also reported [73]. 

### 3.3. MAG

This is a minor but important component of myelin that is expressed only in myelin-forming cells and is involved in the initiation of myelination in the CNS [74, 75]. Microarray studies reported decreased MAG mRNA expression in the DLPFC and the middle temporal gyrus of postmortem schizophrenic brains [58, 62]. In studies using quantitative PCR analysis, decreased MAG mRNA was found in the anterior cingulated cortex and the hippocampus, in addition to DLPFC [59, 76]. These findings were confirmed in a recent study that reported a decrease in the expression of MAG in white matter in schizophrenia using a probe that detected mRNAs for the large and small MAG splice variants. However, expression of MAG did not differ between patients with schizophrenia and controls in the grey or white matter in another study [77]. Discrepancy was also seen in genetic association analyses; some, but not all, of the analyses, linked MAG gene to schizophrenia [78–80]. 

### 3.4. OLIG2

This gene maps to 21q22.11 and encodes a transcription factor central to OL development. Strong association of *OLIG2 *and schizophrenia has been reported. There are reports of deletion in this region in patients with schizophrenia [81] and of a low risk of schizophrenia in people with trisomy 21 [82]. In the postmortem schizophrenic brain, *OLIG2* mRNA reduced [60, 62, 83]. *OLIG2* expression in cerebral cortex significantly correlated with *CNP *and ERBB4, suggesting interaction effects on disease risk between *OLIG2* and *CNP *[84]. 

### 3.5. QKI

In a genome scan of a single large family from northern Sweden with high frequency of schizophrenia and schizophrenia-spectrum disorders, Lindholm et al. [85] detected a maximum LOD (logarithm of data) score of 6.6 on chromosome 6q25. This region contains only one gene described in the literature and the human databases, quaking homolog, KH domain RNA binding (mouse) (*QKI*) [86], pointing to the potential involvement of *QKI* in schizophrenia. In support of this suggestion, expression of *QKI* mRNA decreased in seven cortical regions and the hippocampus in the schizophrenic subjects [87], and relative mRNA expression levels of two *QKI* splice variants clearly downregulated in schizophrenic patients [88]. Moreover, mRNA levels of the tightly coexpressed myelin-related genes including *PLP1, MAG, MBP, TF, SOX10, *and *CDKN1B* decreased in schizophrenic patients, as compared with control individuals. Most of these differences (68–96%) can be explained by variation in the relative mRNA levels of *QKI*-7 kb. The same *QKI* splice variant was shown to be downregulated in patients with schizophrenia. Therefore, the authors suggested that decreased activity of some myelin-related genes in schizophrenia may be caused by disturbed *QKI* splicing [89]. 

### 3.6. The Other Myelin-Related Genes

In addition to the aforementioned genes, there have also been reports of association with schizophrenia for the myelin-oligodendrocyte glycoprotein (*MOG*) [90], the proteolipid protein 1 gene (*PLP1*) [91], and the transferring gene (*TF*) [92]. Of these, *PLP1* warrants to be emphasized here. Proteins (PLP1 and its splicing variant DM20) encoded by this gene are synthesized by OLs as the two major integral proteins of myelin membranes of CNS [93]. Point mutations of human *PLP* have been recognized as the molecular basis of one form of leukodystrophy, the X-chromosome-linked Pelizaeus-Merzbacher disease (PMD). And a novel mutation in the *PLP* gene has been reported to lead to PMD [94]. Lower levels of *PLP1* mRNA have been reported in schizophrenia [59, 62, 95]. There is also evidence for a genetic association of *PLP1* with schizophrenia [91]. However, in Japanese population, no association was found between *PLP1* and schizophrenia [96]. 

## 4. Abnormal Behaviors in Patients with White Matter Diseases

The third line of evidence for the involvement of white matter abnormalities in schizophrenia came from studies reporting abnormal behaviors in human sufferers from white matter diseases. 

### 4.1. Agenesis of Corpus Callosum (ACC)

The corpus callosum is the largest white matter tract in the brain. Two developmental malformations of the corpus callosum associated with psychosis are partial or complete ACC and callosal lipoma [97]. When psychiatric disturbance presents in ACC sufferers, it is psychotic in nature in at least half of the patients [98]. Psychosis is also seen in Andermann's and Apert's syndrome at a higher rate, compared to healthy controls. Both Andermann's and Apert's syndrome are accompanied with ACC [99, 100]. On the other hand, undiagnosed ACC has been detected in schizophrenic patients at a significantly higher rate [101]. 

### 4.2. Metachromatic Leukodystrophy (MLD)

This is a devastating demyelinating disease caused by a deficiency of the enzyme sulfatide sulfatase, also known as arylsulfatase A (ASA). Patients with MLD have abnormalities predominantly in the frontotemporal white matter. Up to 50% of patients with adolescent or early-adult onset present with psychotic symptoms such as auditory hallucinations, though disorder, affective disturbance, formal thought disorder, and catatonia [102]. In many cases of MLD, the behavioral abnormalities are the first symptoms. Some of these forms have been diagnosed as schizophrenia. Very seldom, neurological symptoms, especially ataxia, occur without cognitive or psychiatric disturbances [103]. On the other hand, a large number of adult patients with varying psychiatric manifestations have low levels of ASA-CS activity, suggesting that such patients may be asymptomatic carriers of the sulfatidase defect (heterozygotes for MLD) [104]. 

### 4.3. The Adult Onset Form of Niemann-Pick Type C (NPC) Disease

This is a lipid storage disorder. In the early stage of NPC, only white matter is affected [105, 106]. Patients show white matter disruption in the corpus callosum [107] and periventricular white matter [108]. Up to 40% of cases, a rate comparable with MLD, present initially with psychosis [108–112]. 

### 4.4. Multiple Sclerosis (MS)

This is a demyelinating disease of CNS. The onset of most cases occurs between 20 and 40 years of age [113], reminiscent of the onset of schizophrenia that occurs mainly between the ages of 16 and 30 years [2]. In addition to the cardinal pathological features of focal areas of demyelination and immune-mediated inflammation, patients with MS show a number of different behavioral syndromes, which may be broadly divided into two categories: those pertaining to mood, affect, and behavior and those impairing cognitive functions [114]. Recent epidemiological studies estimated that the prevalence of psychosis in MS patients is two to three times those in the general population [115]. More interestingly, the prevalence is the highest (about 4.2%) in the 15- to 24-year age group in MS patients, again, which reminds us of the early onset of schizophrenia. 

In patients with clinically definite MS, cognitive abnormalities can be detected in 40–60% of patients [116]. Memory and executive functions are often impaired to an extent that cannot be explained as a result of the general intellectual decline [117]. Moreover, impairment in sustained attention, processing speed, and verbal memory in MS patients negatively correlated with MS lesion volume in frontal and parietal regions at baseline, 1-year, and 4-year followup, suggesting a contribution of the frontoparietal subcortical networks disruption to these cognitive impairments in MS [118]. 

## 5. Oligodendrocyte-Related Genetic Animal Models of Schizophrenia

Although a number of biologically related genes have been reported to be downregulated in schizophrenia as reviewed above, only a few genetic animal models have been reported that show white matter development disruption and schizophrenia-related behaviors thus being used as potential animal models of schizophrenia. 

### 5.1. Plp1 Transgenic Mice Show Schizophrenia-Related Behaviors

The first animal study that showed both white matter development disruption and abnormal behaviors was done by Boison and Stoffel [119]. They produced transgenic mice carrying a target alteration of the *plp* gene containing a deletion within exon III, mimicking DM20, and a neocassette in reverse orientation within intron III. The ultrastructure of the multilayer myelin sheath of all axons in the CNS of hemizygous male or homozygous female PLP/DM20-deficient mice is highly disordered. This disrupted assembly of the myelin sheath was accompanied with profound reduction of conductance velocity of CNS axons, impairments in neuromotor coordination, and reduced spontaneous locomotor activity. In a more recent study, Tanaka et al. [120] analyzed a transgenic mouse line harboring extra copies of the *plp1* gene (*plp*1^tg/−^ mice) at 2 months of age. Although the *plp*1^tg/−^ mice showed an unaffected myelin structure, the conductance velocity in all axonal tracts tested in the CNS greatly reduced. Moreover, the *plp*1^tg/−^ mice showed altered anxiety-like behaviors, reduced prepulse inhibitions (PPI), spatial learning deficits and working memory deficit. These abnormal behaviors are schizophrenia-related behaviors, suggesting that the *plp*1^tg/−^ mice may be used as a potential animal model to examine the role of altered *plp1* gene in schizophrenia. 

### 5.2. Functional Consequence of Perturbing NRG1/erbB4 Signaling

In the functional studies, mutant mice heterozygous for either *NRG1* or its receptor erbB4 showed a behavioral phenotype that overlaps with mouse models for schizophrenia. Furthermore, *NRG1* hypomorphs had fewer functional NMDA receptors than wild-type mice. More interestingly, the behavioral phenotypes of the *NRG1* hypomorphs were partially reversible with clozapine, an atypical antipsychotic drug used to treat schizophrenia [63]. Since then a number of mutant mice with heterozygous deletion of transmembrane domain NRG1 have been replicated in independent laboratories [121], including hyperactivity in a novel environment [122, 123], mild disruption of PPI [124], and social interaction deficits [123, 125]. But both spatial learning and memory, assessed in the Barnes maze, and spatial working memory, as measured by nondelay Y-maze alternation, kept intact [123]. Similarly, there was no effect of *NRG1* genotype on performance in either test of emotionality/anxiety [125]. 

To test whether erbB signaling contributes to psychiatric disorders by regulating the structure or function of OLs, Roy et al. [126] analyzed transgenic mice in which erbB signaling was blocked in OLs *in vivo*. Loss of erbB signaling led to changes in OL number and morphology, reduced myelin thickness, and slower conduction velocity in CNS axons. Furthermore, these transgenic mice exhibited increased levels of DA receptors and transporters and behavioral alterations including reduced locomotion and social dysfunction. More interestingly, *BACE1 *(*β*-site APP-cleaving enzyme 1) knockout mice, in which *NRG1* processing was altered, exhibited deficits in PPI, novelty-induced hyperactivity, hypersensitivity to a glutamatergic psychostimulant (MK-801), cognitive impairments, and deficits in social recognition. Some of these manifestations were responsive to clozapine, an atypical antipsychotic drug. Although the total amount of ErbB4 did not change in *BACE1 *knockout mice, binding of erbB4 with postsynaptic density protein 95 significantly reduced in the brains of these mice [127]. Together, the above studies suggest that altered *NRG1*/erbB4 signaling plays an important role in the pathogenesis of schizophrenia. 

### 5.3. Nogo-A Deficient Mice

In addition to the above two animal models, a mouse model of constitutive genetic Nogo-A deficiency deserves to be emphasized here. In a comprehensive series of behavioral tests with specific relevance to schizophrenia pathopsychology, the Nogo-A deficient mice showed deficient sensorimotor gaiting, disrupted latent inhibition, perseverative behavior, and increased sensitivity to the locomotor stimulating effects of amphetamine. Moreover, these behavioral changes were accompanied by altered monoaminergic transmitter levels in specific striatal and limbic structures, as well as changes in D_2_ receptor expression in the same brain regions [128]. Therefore, the authors concluded that Nogo-A may bear neuropsychiatric relevance, and alterations in its expression may be one etiological factor in schizophrenia and related disorders. 

## 6. Cuprizone-Fed Mouse: A Novel Animal Model of Schizophrenia

### 6.1. A Murine Model of Demyelination/Remyelination

Cuprizone (CPZ: biscyclohexanone oxalyldihydrazone) is a copper chelator used as a reagent for copper analysis. In early studies [129, 130], higher doses (0.3, 0.5, and 0.75%) of CPZ were administered to animals via diet. These treatments were extremely toxic to mice, manifested with severe growth reduction, posterior paresis, and high mortality early in the feeding period. Convulsion and seizures were also seen in later stages (6-7 weeks after CPZ-feeding). Pathological alterations include severe status spongiosus, astrogliosis, demyelination, and hydrocephalus. Under electron microscopy, there are many large vacuoles within the myelin sheaths and swollen glial cells. The vacuoles, which resulted from giant mitochondria, were also seen in the hepatocytes [129]. Later studies [131, 132] administered a lower dose (0.2%) of CPZ to mice. The animals have no evident toxic effects and neurological symptoms; consistent demyelination and mature OLs loss are main pathological alterations. When allowed to recover on a normal diet, remyelination begin within a week and progresses until all axons are myelinated. For these features, the CPZ models have been extensively used to define issues important to understanding of the pathophysiology of demyelination and to gain understanding of the mechanisms involved in remyelination. 

### 6.2. Behavioral Deficits in CPZ-Fed Mouse

Given that demyelination and mature OLs loss are main pathological alterations in brains of mice exposed to the lower dose (0.2%) of CPZ, examining possible behavioral deficits in the CPZ-fed mice should provide informative data relating specific behaviors to regional white matter abnormality. In the first report by Liebetanz and Merkler [133], central motor deficits were observed in mice fed the CPZ-containing diet by using a novel murine motor test, the motor skill sequence. This test was designed to detect latent deficits in motor performance. In a first step, mice were habituated to training wheels composed of regularly spaced crossbars till maximal wheel-running performance was achieved. Then, the animals were exposed to wheels with irregularly spaced crossbars demanding high-level motor coordination. Demyelinated mice showed reduced running performance on the training wheels as compared to controls. This deficit was even more pronounced when these mice were subsequently exposed to the complex wheels. Interestingly, remyelinated animals after CPZ withdrawal showed normal performance on the training wheels but abnormal performance on the complex wheels. 

The poor motor coordination of the CPZ-fed mice was also detected in the rota-rod analysis [134]. In addition, in the 3rd and 4th weeks after 0.2% CPZ treatment, the mice exhibited an increase in CNS activity, that is, an increase in climbing during the functional observation battery (FOB) tests, and an inhibited anxiogenic response to the novelty challenge (open-field) test. The FOB protocol consisted of 18 endpoints which evaluate CNS activity and excitability, neuromuscular and autonomic effects, and sensorimotor reactivity [135]. The results related white matter abnormality to emotional behavior and reminded us of the previous finding that transection of the rat's corpus callosum induces an increased number of rearing and activity in the centre of the open field [136, 137]. 

### 6.3. A Novel Animal Model of Schizophrenia

In 2008, we, for the first time, examined effects of quetiapine, an atypical antipsychotic drug, on OLs [138]. We started from the *in vitro* effects of quetiapine on OL development. Quetiapine was shown to increase the proliferation of neural progenitor cells (NPCs) in the presence of growth factors, direct the differentiation of NPCs into OL lineage through extracellular signal-related kinases, upregulate the expression of MBP, and stimulate the myelination of axons by OLs in rat embryonic neocortical aggregate cultures. In the last experiment of this study, chronic administration of quetiapine prevented the CPZ-induced myelin breakdown and spatial working memory impairment in C57BL/6 mice. This protective effect of quetiapine on the CPZ-induced white matter abnormality was further substantiated in a following animal study. This drug dramatically decreased the numbers of activated microglia and astrocytes teemed in demyelinated sites, in addition to ameliorating myelin breakdown and MBP decrease in the brain [139]. 

Inspired by the above studies, we further characterized the behavioral and neurobiological changes in the CPZ-fed mice [140]. Mice exposed to CPZ for 2 and 3 weeks displayed more climbing behavior and PPI deficits. In addition, they showed lower activities of monoamine oxidase (MAO) and DA beta hydroxylase (DBH) in the hippocampus and PFC and had higher DA but lower NE levels in PFC. Mice exposed to CPZ for 4 to 6 weeks, when demyelination, myelin breakdown, and OL loss were evident, showed less social interaction compared to controls. At all time points, the CPZ-exposed mice spent more time in the open arms of an elevated plus-maze and exhibited spatial working memory impairment. The social interaction decrease and spatial working memory impairment were also reported in an independent study from other investigators [141]. These abnormal behaviors are reminiscent of some schizophrenia symptoms seen in human patients, thus suggested that the CPZ-fed mouse may be used as a novel animal model of schizophrenia to explore roles of white matter abnormalities in the pathophysiology and treatment of this mental disorder. 

**Figure 1:**
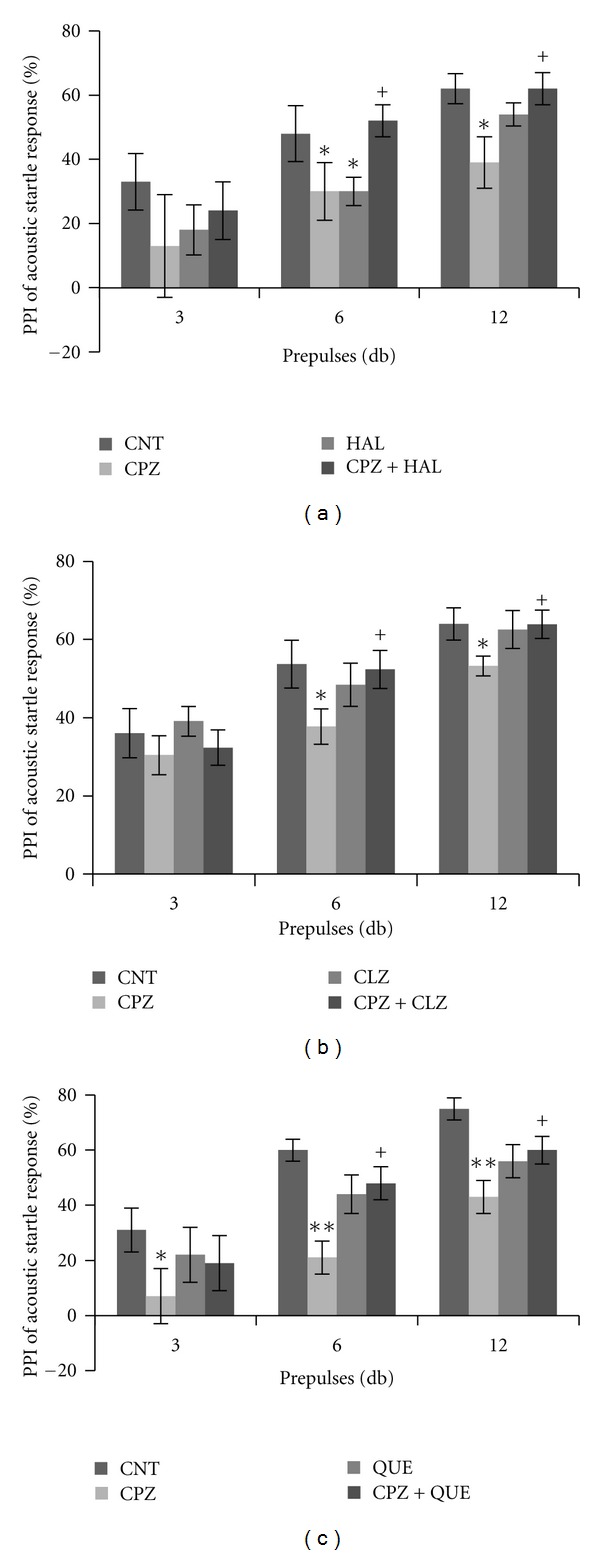
Effects of antipsychotics on the CPZ-induced deficits in PPI test. Control and experimentally treated C57BL/6 mice were subjected to PPI test on the same day (14th day after CPZ exposure). (a) The data of the HAL experiment. (b) The data of the CLZ experiment. (c) The data of the QUE experiment. Data were expressed as M ± SEM (*n* = 6 to 12/group). CNT: control group; CPZ: cuprizone group; CLZ: clozapine group; HAL: haloperidol group; QUE: quetiapine group; CPZ+CLZ: mice received both cuprizone and clozapine; CPZ+HAL: mice received both cuprizone and haloperidol; CPZ+QUE: mice received both cuprizone and quetiapine. PPI: prepulse inhibition; db: decibel. *P < 0.05, **P < 0.01, compared to the CNT group; ^+^ *P* < 0.05, compared to the CPZ group.

**Figure 2:**
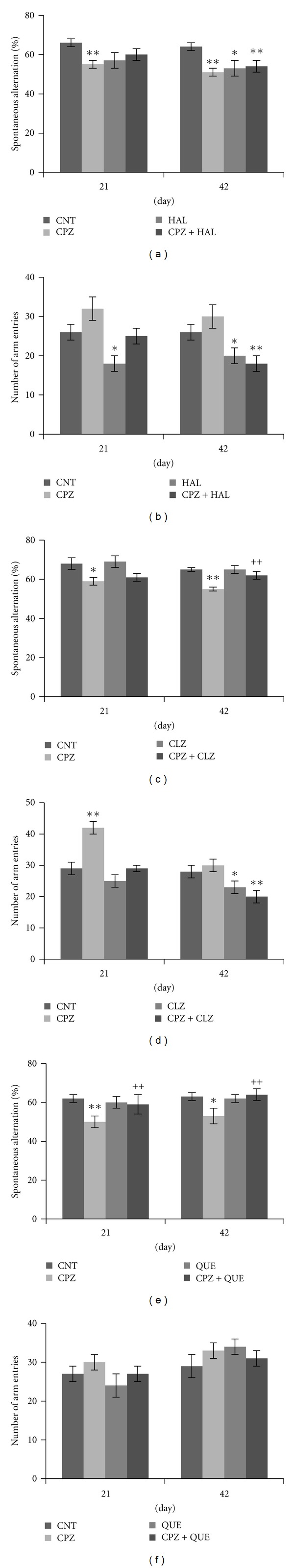
Effects of antipsychotics on the CPZ-induced abnormal performance in the Y-maze test. Control and experimentally treated C57BL/6 mice were subjected to Y-maze test on the same days (21st and 42nd days after CPZ exposure). (a) The data of the spontaneous alternation in the HAL experiment. (b) The data of the number of arm entries in the HAL experiment. (c) The data of the spontaneous alternation in the CLZ experiment. (d) The data of the number of arm entries in the CLZ experiment. (e) The data of the spontaneous alternation in the QUE experiment. (f) The data of the number of arm entries in the QUE experiment. Data were expressed as M ± SEM (*n* = 6 to 12/group). *P < 0.05, **P < 0.01, compared to the CNT group; ^++^ *P* < 0.01, compared to the CPZ group.

**Figure 3:**
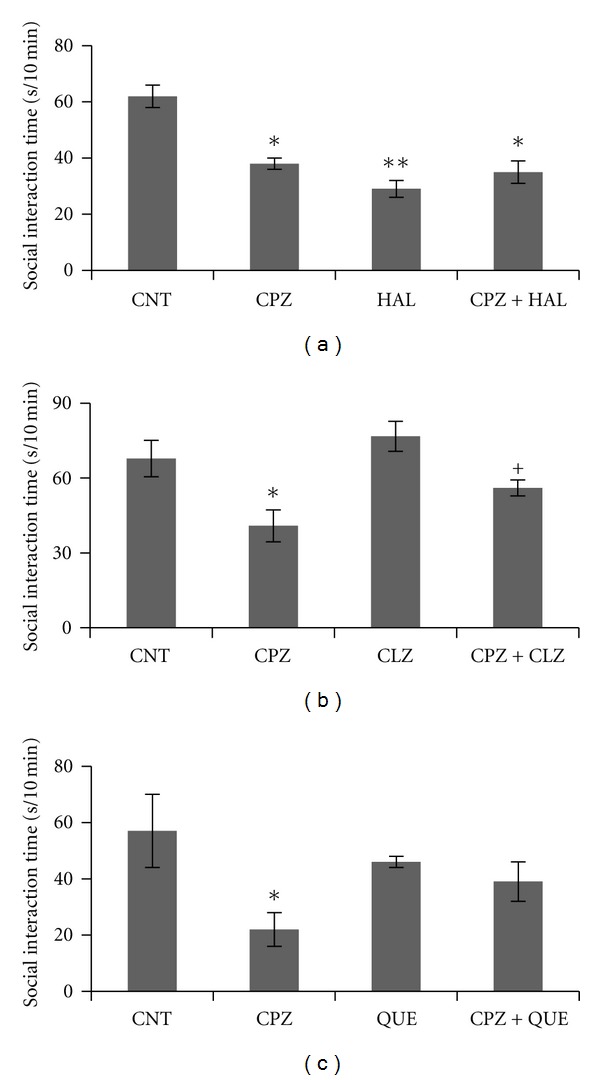
Effects of antipsychotics on the CPZ-induced deficits in the social interaction. Control and experimentally treated C57BL/6 mice were subjected to social interaction test on the same day (28th day after CPZexposure). (a) The data of the HAL experiment. (b) The data of the CLZ experiment. (c) The data of the QUE experiment. Data were expressed as M ± SEM (*n* = 6 pairs/group). *P < 0.05, **P < 0.01, compared to the CNT group; ^+^ *P* < 0.05, compared to the CPZ group.

**Figure 4:**
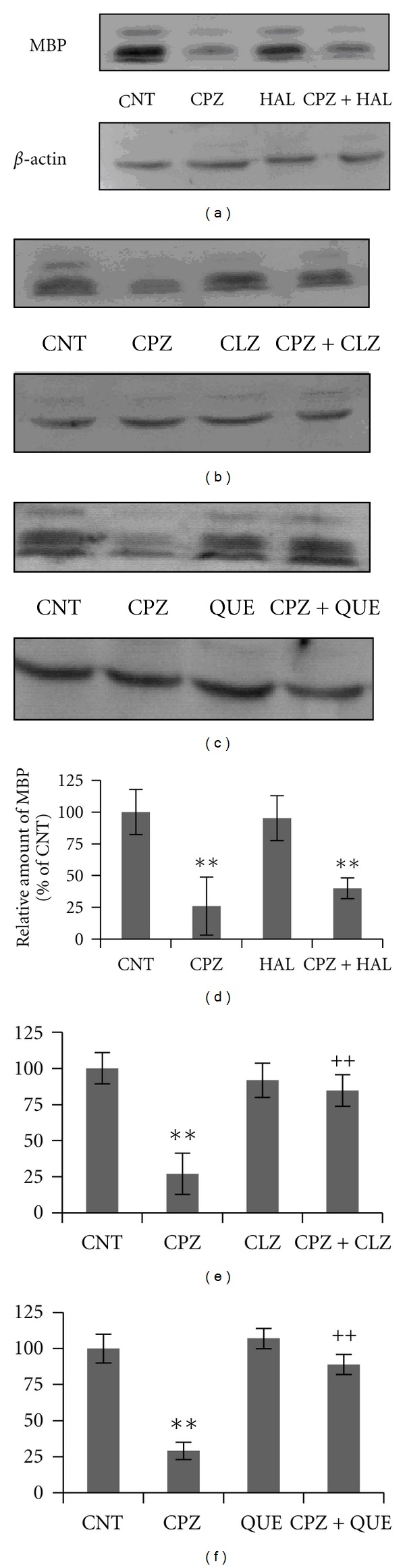
Effects of antipsychotics on the CPZ-induced decrease in MBP in CP. Control and experimentally treated C57BL/6 mice were sacrificed on 43rd day after CPZ exposure. The CP was dissected out of the brain and processed for Western-blot analysis to measure MBP levels. The upper photographs (a), (b), and (c) are representative of Western blots from the HAL, CLZ, and QUE experiments, respectively. The bar charts (d), (e), and (f) in the bottom panel are the statistical results of the amount of MBP relative to *β*-actin in the same corresponding lanes as labeled. Data were expressed as M ± SEM (*n* = 6/group). **P < 0.01, compared to the CNT group; ^++^ *P* < 0.01, compared to the CPZ group.

More significantly, the CPZ-induced behavioral changes showed different responses to typical and atypical antipsychotics [142]. All tested antipsychotics (haloperidol, clozapine, and quetiapine), when coadministered with CPZ to mice, effectively blocked the PPI deficits (Figure 1); clozapine and quetiapine, but not haloperidol, prevented CPZ-fed mice from spatial working memory impairment (Figure 2); clozapine and quetiapine, but not haloperidol, ameliorated social interaction decrease (Figure 3). These different effects of typical and atypical antipsychotics on abnormal behaviors seem to be related to their effects on the CPZ-induced white matter abnormalities as clozapine and quetiapine, but not haloperidol, ameliorated the myelin breakdown and MBP decrease in PFC, hippocampus, and caudate putamen (Figure 4). These results provide experimental evidence for the protective effects of antipsychotics on white matter abnormalities and the concurrent behavioral changes in CPZ-fed mice. 

In a more recent study, we observed the time courses of behavioral abnormalities and remyelination in mice after CPZ withdrawal and examined effect of antipsychotics on the recovery processes [143]. The CPZ-induced abnormal performance on the elevated plus-maze recovered to the normal range within two weeks after CPZ withdrawal. In contrast, alterations in social interaction showed no recovery within the three-week postwithdrawal recovery period. And the social interaction deficit did not respond to any one of the antipsychotics (clozapine, haloperidol, olanzapine, and quetiapine) tested in this study. Altered performance in the Y-maze showed some recovery in the vehicle group; clozapine, olanzapine, and quetiapine, but not haloperidol, significantly promoted this recovery process. None of the drugs affected the recovery of damaged white matter within the three-week recovery period. These ineffective results may be due to inappropriate doses of tested drugs in this study and/or reflect the intractable feature of these abnormalities. In the latter case, a reasonable suggestion would be that the damage to OL/myelin in early phase could leave permanent damage on neural connectivity and/or its functions. To test this hypothesis, future studies should investigate the remyelination and functional recovery processes in longer recovery periods by means of various experimental approaches including electron microscopy and electrophysiological techniques. 

While most of animal studies applied CPZ to C57BL/6 mice, efforts were also made to develop a rat model of demyelination in the CNS. After exposed to CPZ for two and four weeks, rats showed a decrease in mRNA transcripts and protein levels of OL-specific genes in PFC [144]. Levels of myelin-related genes did not change in the striatum and hippocampus, two brain areas that should have been completely myelinated before the age of CPZ exposure. In addition, glial fibrillary acidic protein upregulated in PFC, indicating an activation of astrocytes. More interestingly, rats treated for two weeks with CPZ showed an increased difficulty to shift attention from one perceptual dimension to another in the extra dimensional shift phase of the attention set-shifting task, a modified version of the Wisconsin Card Sorting Test which depend on PFC [145]. Importantly, CPZ-treated rats did not exhibit locomotor problems and had normal weight gain. Thus, the CPZ rat model can be used to study developmental vulnerability of white matter, as well as the pathogenesis and behavioral consequences of dysmyelination [146]. 

### 6.4. Comparing CPZ Model with Genetic Models of Schizophrenia

To summarize the aforementioned animal models of schizophrenia, Table 1 listed the main observations done so far on these animal models. Of the transgenic animal models, the NRG1-erbB4 transgenic mouse seems to have more similarities to observations in patients with schizophrenia, while relatively few studies have been done in the other two genetic animal models. However, studies have shown that both hypermorphic [147, 148] and hypomorphic [149–151] expression of the NRG1 gene may produce several common behavioral phenotypes in animals, which warrants further studies to address the underlying mechanisms on these behavioral abnormalities. The CPZ-fed mouse provided an alternative animal model showing white matter abnormalities in the brain- and schizophrenia-related behaviors. More interestingly, these CPZ-induced changes differently responded to haloperidol and atypical antipsychotic drugs. 

## 7. Concluding Remarks

There is a great body of literature reporting white matter abnormalities in patients with schizophrenia, including imaging and postmortem evidence, suggesting a putative role of altered white matter in schizophrenia. Given its role as the primary infrastructure for long-distance communication in the brain, the evidence of altered white matter is agreeable to the disconnectivity theory of schizophrenia that emphasizes the role of abnormal interactions between brain regions [152]. Now, OLs and myelin dysfunction have been linked to neurocircuitry abnormalities in schizophrenia [153]. 

The white matter implication hypothesis can account for why the symptoms of schizophrenia commonly first present in late adolescence and early adulthood as the white matter development is still going on during these periods [13]. It is also consistent with the neurodevelopmental theory of schizophrenia [154]. According to this theory, the interaction of genetic vulnerability and early environmental exposures can induce a developmental trajectory which culminates later in the clinical syndrome [154, 155]. Indeed, molecular genetics analyses revealed altered OL- and myelin-related genes in schizophrenia. 

Another line of evidence supporting the white matter implication hypothesis in schizophrenia came from patients with white matter diseases. As illustrated by ACC, MLD, NPC, and MS, white matter lesions are commonly accompanied with certain schizophrenia symptoms. These human studies warrant future experimental studies to examine a putative role of altered white matter in schizophrenia.

The use of transgenic and mutant animal models offers a unique opportunity to analyze OLs and relevant changes in schizophrenia [156]. Examples included in this paper are the *plp1 *transgenic mice, mutant mice heterozygous for either *NRG1* or its receptor erbB4, and the Nogo-A deficient mice. These transgenic and mutant mice show both white matter development disruption and schizophrenia-related behaviors thus may be used as potential animal models of schizophrenia. More informative data are expected to come from further studies with these animal models. 

Although CPZ is a neurotoxic compound, the white matter alterations seen in CPZ-fed mice are not conflict with the neurodevelopment theory of schizophrenia. Indeed, genetic and developmental factors, such as animal species, age, and developmental status of a white matter structure, have significant impacts on the white matter alterations in CPZ-fed animals [141, 143, 157]. Moreover, the abnormal behaviors seen in CPZ-fed mice and rats [140, 141, 143] are reminiscent of schizophrenia symptoms including positive and negative symptoms as well as cognitive impairment. In addition, CPZ-fed mice show higher DA levels in PFC and lower NE levels in the same brain region. These changes in DA and NE may account for the abnormal climbing behavior and PPI deficits occurred before the appearance of demyelination and myelin breakdown [140]. High levels of DA in PFC may also contribute to demyelination and myelin breakdown in this brain region. This notion is in accordance with the finding that chronic administration of amphetamine (1.0 mg/kg) to mice caused microstructural changes in the white matter of frontal cortex and induced higher locomotion and spatial working memory impairment [158]. The abnormal white matter, in turn, may affect DA neurotransmission in the brain and thus cause behavioral changes. In line with this view, a new hypothesis of schizophrenia has been proposed, which theorized that the abnormal myelination of late-developing frontal white matter is a single underlying cause of the three distinctive features of this disorder, namely, its excessive DA neurotransmission, its frequent periadolescent onset, and its bizarre, pathognomonic symptoms [159]. Extensive studies are necessary to further address the relationship of excessive DA neurotransmission and abnormal myelination in schizophrenia. 

## Figures and Tables

**Table 1:** Comparison of animal models examining a putative role of altered white matter in schizophrenia.

	plp1^tg/−^ mice	NRG1-erbB4 transgenic mice	Nogo-A deficient mice	CPZ-fed mice
Altered myelin structure	—	*√*	No data	*√*
OL loss	No data	*√*	No data	*√*
Decreased axonal conductance	*√*	*√*	No data	No data
Dopamine level	No data	No data	↓	↑
D_2_ receptors	No data	↑	↓	No data
NMDA receptors	No data	↓	No data	No data
Hyperactivity	No data	*√*	No data	*√*
Anxiety-like behaviors	*√*	*√*	*√*	*√*
Disrupted PPI	*√*	*√*	*√*	*√*
Spatial learning deficit	*√*	*√*	No data	*√*
Working memory deficit	*√*	*√*	No data	*√*
Impaired social activities	No data	*√*	—	*√*
Response to antipsychotics	No data	*√*	No data	*√*
